# Role of Permissive Neuraminidase Mutations in Influenza A/Brisbane/59/2007-like (H1N1) Viruses

**DOI:** 10.1371/journal.ppat.1002431

**Published:** 2011-12-08

**Authors:** Yacine Abed, Andrés Pizzorno, Xavier Bouhy, Guy Boivin

**Affiliations:** Research Center in Infectious Diseases of the CHUQ-CHUL and Laval University, Québec City, Québec, Canada; University of Wisconsin-Madison, United States of America

## Abstract

Neuraminidase (NA) mutations conferring resistance to NA inhibitors were believed to compromise influenza virus fitness. Unexpectedly, an oseltamivir-resistant A/Brisbane/59/2007 (Bris07)-like H1N1 H275Y NA variant emerged in 2007 and completely replaced the wild-type (WT) strain in 2008–2009. The NA of such variant contained additional NA changes (R222Q, V234M and D344N) that potentially counteracted the detrimental effect of the H275Y mutation on viral fitness. Here, we rescued a recombinant Bris07-like WT virus and 4 NA mutants/revertants (H275Y, H275Y/Q222R, H275Y/M234V and H275Y/N344D) and characterized them *in vitro* and in ferrets. A fluorometric-based NA assay was used to determine *Vmax* and *Km* values. Replicative capacities were evaluated by yield assays in ST6Gal1-MDCK cells. Recombinant NA proteins were expressed in 293T cells and surface NA activity was determined. Infectivity and contact transmission experiments were evaluated for the WT, H275Y and H275Y/Q222R recombinants in ferrets. The H275Y mutation did not significantly alter *Km* and *Vmax* values compared to WT. The H275Y/N344D mutant had a reduced affinity (*Km* of 50 vs 12 µM) whereas the H275Y/M234V mutant had a reduced activity (22 vs 28 U/sec). In contrast, the H275Y/Q222R mutant showed a significant decrease of both affinity (40 µM) and activity (7 U/sec). The WT, H275Y, H275Y/M234V and H275Y/N344D recombinants had comparable replicative capacities contrasting with H275Y/Q222R mutant whose viral titers were significantly reduced. All studied mutations reduced the cell surface NA activity compared to WT with the maximum reduction being obtained for the H275Y/Q222R mutant. Comparable infectivity and transmissibility were seen between the WT and the H275Y mutant in ferrets whereas the H275Y/Q222R mutant was associated with significantly lower lung viral titers. In conclusion, the Q222R reversion mutation compromised Bris07-like H1N1 virus *in vitro* and *in vivo*. Thus, the R222Q NA mutation present in the WT virus may have facilitated the emergence of NAI-resistant Bris07 variants.

## Author Summary

The H275Y neuraminidase (NA) mutation conferring resistance to oseltamivir was shown to impair old influenza H1N1 strains both *in vitro and in vivo*. By contrast, an oseltamivir-resistant A/Brisbane/59/2007 (Bris07)-like H1N1 H275Y NA variant emerged in 2007 and completely replaced the wild-type (WT) strain in 2008–2009. This discrepancy could be attributed to permissive NA mutations (R222Q, V234M and D344N) that were identified in most Bris07-like oseltamivir-resistant variants. To verify this hypothesis, we developed a reverse genetics system for a sensitive Bris07-like isolate (275H) whose NA protein contains the 3 permissive mutations (222Q, 234M, 344N). Using mutagenesis, we first introduced the H275Y then reverted codons at positions 222, 234 and 344. The resulting 5 recombinants (WT, H275Y, H275Y/Q222R, H275Y/M234V and H275Y/N344D) were compared with regard to NA enzyme properties, replicative capacities *in vitro* as well as infectivity and contact-transmissibility in ferrets. Among the studied permissive mutations, Q222R was associated with a significant reduction of both affinity and activity of the NA enzyme resulting in a virus with a reduced replicative capacity *in vitro* and decreased replication in lungs of ferrets. Thus, the R222Q mutation may have been the major permissive NA change that facilitated the emergence and spread of NAI-resistant Bris07 variants.

## Introduction

Influenza viruses are respiratory pathogens associated with significant public health consequences. Each year, influenza epidemics can be responsible for significant morbidity in the general population and excess mortality in elderly patients and individuals with chronic underlying conditions. Influenza A viruses of the H1N1 subtype have been associated with seasonal influenza epidemics for many decades and, in presence of immunological pressure, such viruses continue to evolve through genetic variability which is mainly confined to virus segments encoding surface glycoproteins i.e., the hemagglutinin (HA) and neuraminidase (NA) [1]. Consequently, viral strains to be used in annual influenza vaccines should be regularly updated to ensure optimal protection. Besides vaccines, neuraminidase inhibitors (NAI) including inhaled zanamivir, oral oseltamivir and intravenous peramivir provide an important additional measure for the control of influenza infections [2]. These antivirals target the active center of the influenza NA molecule, which is constituted by 8 functional (R-118, D-151, R-152, R-224, E-276, R-292, R-371, and Y-406; N2 numbering) and 11 framework (E-119, R-156, W-178, S-179, D-198, I-222, E-227, H-274, E-277, N-294, and E-425; N2 numbering) residues that are largely conserved among influenza A and B viruses [3]. However, the emergence of NAI-resistant viruses, as a result of drug use or due to circulation of natural variants, may compromise the clinical utility of this class of anti-influenza agents.

The H275Y (H274Y in N2 numbering) NA mutation conferring resistance to oseltamivir and peramivir has been detected with increasing frequency in seasonal A/H1N1 viruses since 2007 to the extent that almost all characterized A/Brisbane/59/2007-like (Bris07) (H1N1) influenza strains that circulated worldwide during the 2008–09 season were H275Y variants [4], [5]. Interestingly, this drug-resistant strain seemed to have emerged independently of NAI use [6], [7]. The rapid dissemination of the H275Y Bris07 variants in the absence of antiviral pressure suggests that the H275Y NA mutation may not compromise viral fitness and transmissibility in this recent H1N1 viral background. This contrasts with previous studies that analyzed the role of the H275Y mutation using older (A/Texas/36/91 [8] and A/New Caledonia/99/01 [9]) drug-selected H1N1 variants. Recent reports by our group and others have confirmed the differential impact of the H275Y mutation on viral fitness and enzymatic properties in the context of old and recent influenza H1N1 isolates [10], [11]. In an attempt to provide a molecular explanation for this observation, previous authors suggested that secondary NA mutations such as D344N that emerged in H1N1 variants isolated after the 2006–07 season were associated with higher NA activity and affinity and could have facilitated the emergence of the H275Y mutation [11], [12]. Such drug-resistant mutants may have a better HA-NA balance than the susceptible viruses and indeed completely replaced them in a short period of time. In addition, Bloom and colleagues recently described two other secondary NA mutations at codons 222 and 234 that may have counteracted the compromising impact of the H275Y mutation [13]. In that study, the V234M and R222Q mutations were shown to restore the viral fitness of an A/New Caledonia/20/99 H1N1 variant containing the H275Y mutation [13].

To further investigate which secondary NA mutations may have facilitated the introduction of the H275Y mutation in contemporarily seasonal H1N1 viruses and allowed their dissemination, we developed a reverse genetics system using a clinical Bris07 (H1N1) isolate as genetic background and evaluated the impact of the H275Y oseltamivir resistance mutation as well as several potential compensatory NA mutations on enzyme activity, viral fitness and transmissibility.

## Results

**Figure 1:**
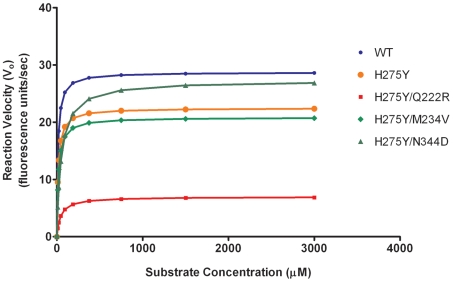
Neuraminidase (NA) enzyme kinetics of recombinant A/Brisbane/59/2007-like (H1N1) viruses. The rate of substrate conversion velocity (V_0_) by NA enzymes from a standardized dose of 10^6^ PFU/ml of recombinant virus was determined. The fluorogenic substrate (MUNANA) was used at final concentrations of 0 to 3000 µM. Fluorescence was measured every 90 sec for 53 min at 37 °C using excitation and emission wavelengths of 355 and 460 nm, respectively. The data of one representative experiment performed in triplicate is shown.

In the present study, five recombinant Bris07 influenza viruses were generated i.e., the WT virus (containing the putative permissive mutations) that briefly circulated during the 2007–08 season, the single H275Y oseltamivir-resistant variant and three double mutants containing the H275Y mutation as well as reversion of potential permissive mutations (H275Y/Q222R, H275Y/M234V and H275Y/N344D). NA enzymatic properties using equivalent titers of recombinants were first analyzed with determination of relative NA enzymatic activity (*Vmax* values), which reflects the total NA activity per virion, and *Km* values, which reflect the affinity for the substrate. As shown in Table 1, the single H275Y mutation had no significant impact on NA affinity and activity compared to the WT virus in the context of the Bris07 background. By contrast, the double H275Y/Q222R mutation was associated with a significant reduction of both NA affinity (*Km* of 40.31 vs 11.95 µM, *P<0.001*) and relative NA activity (7.01 vs 28.19 U/sec, *P<0.001*) compared to the WT (Table 1 and Fig. 1). The H275Y/M234V mutant had a *Km* value comparable to that of the WT, whereas its relative NA activity was significantly reduced (*Vmax* of 21.89 vs 28.19 U/sec, *P<0.05*). The H275Y/N344D mutant showed a significantly reduced affinity (*Km* of 50.77 vs 11.95 µM, *P<0.001*) with no change in NA activity compared to the WT. When comparing the double mutants to the single H275Y mutant, the *Km* values were significantly increased for the H275Y/Q222R and H275Y/N344D mutants (*P<0.001*) whereas only the double H275Y/Q222R mutant had a significantly lower relative NA activity (*P<0.001*).

**Table 1:** Neuraminidase enzymatic properties and plaque areas of recombinant A/Brisbane/59/2007-like (H1N1) viruses.

Recombinants	*Km* a (µM)	*Relative NA activity (Vmax)* a ^(U/sec)^	*Vmax* ratio vs WT	*Plaque area (mm^2^)* b
**WT**	11.95±2.4	28.19±0.74	1.00	0.53±0.17
**H275Y**	16.92±2.25	23.81±1.95	0.84	0.50±0.16
**H275Y/Q222R**	40.31±5.5 ***	7.01±0.11***	0.25	0.13±0.06 ***
**H275Y/M234V**	18.18±1.2	21.89±1.24*	0.78	0.49±0.13
**H275Y/N344D**	50.77±1.0 ***	24.96±2.48	0.89	0.50±0.15

a Values indicate mean *Km* and relative NA activity (*Vmax)* values of a representative experiment performed in triplicate ± standard deviations (SD).

b Values indicate mean plaque area (N = 16) ± SD.

*P<0.05,

***P<0.001 compared to WT.

**Figure 2:**
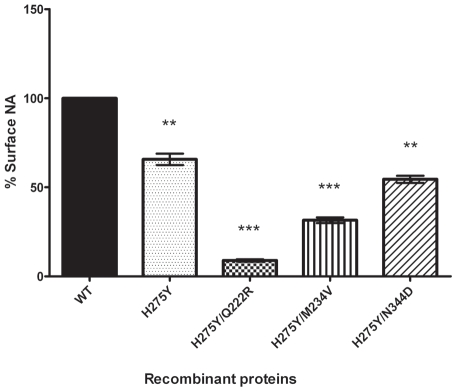
Surface activity of recombinant A/Brisbane/59/2007-like (H1N1) neuraminidase proteins. 293T cells were transfected with pCAGGS-PA, -PB1, -PB2, and -NP plasmids in addition to plasmids expressing the WT or mutant A/Brisbane/59/2007-like neuraminidases (NA) proteins. At 24 h post-transfection, cells were treated with a non-lysing buffer and surface NA activity was measured by using the fluorogenic substrate (MUNANA). Percent surface NA activities were determined in triplicate experiments ± standard deviations. **P<0.01 and ***P<0.001 compared to the WT surface NA activity.

Using recombinant NA proteins expressed in 293T cells, we further investigated the impact of NA mutations on the amount of NA activity at the cell surface. As shown in Fig. 2, all studied mutations were associated with a significant reduction of total surface NA activity compared to the WT with relative total surface activities of 66% (*P<0.01*), 9.72% (*P<0.001*), 32.07% (*P<0.001*) and 54.89% (*P<0.01*) for the H275Y, H275Y/Q222R, H275Y/M234V and H275Y/N344D mutant proteins, respectively. When compared to the single H275Y mutant, H275Y/Q222R (*P<0.001*), H275Y/M234V (*P<0.001*) and H275Y/N344D (*P<0.05*) double mutants also had significantly reduced surface NA activities. The differences observed in total surface NA activity between the different recombinant NA proteins may be due to a decreased number of NA molecules that reached the cell surface or to less activity per enzyme.

We next determined the phenotype of resistance to NAIs for the 5 recombinant viruses. As expected, the presence of the H275Y mutation was associated with resistance to oseltamivir (mean fold increase of 2627 in IC_50_ values) and peramivir (mean fold increase of 998) with no impact on zanamivir susceptibility (Table 2). Interestingly, comparison of the levels of resistance for the double recombinant mutants versus the single H275Y mutant revealed a significant reduction in the level of resistance to peramivir for the double H275Y/Q222R mutant (IC_50_ of 35.25 nM vs 59.85 nM, *P<0.01*). A similar trend was observed for oseltamivir (IC_50_ of 651.86 nM vs 1024.54 nM) although, in this case, the difference between IC_50_ values was not statistically significant.

**Table 2:** Susceptibility profiles of recombinant A/Brisbane/59/2007-like (H1N1) viruses against neuraminidase (NA) inhibitors as assessed by MUNANA NA inhibition assays.

Recombinants	Oseltamivir^a^ (nM)	Zanamivir^a^ (nM)	Peramivir^a^ (nM)
WT	0.39±0.02	0.18±0.03	0.06±0.01
H275Y	1024.54±114.10	0.27±0.06	59.85±2.42
H275Y/Q222R	651.86±116.88	0.18±0.04	35.25±2.11**
H275Y/M234V	1038.56±116.09	0.20±0.01	47.87±1.33
H275Y/N344D	735.07±67.91	0.28±0.03	47.15±3.76

a Values indicated mean IC_50_ values of three experiments ± standard deviations.

**P<0.01 compared to the single H275Y mutant.

**Figure 3:**
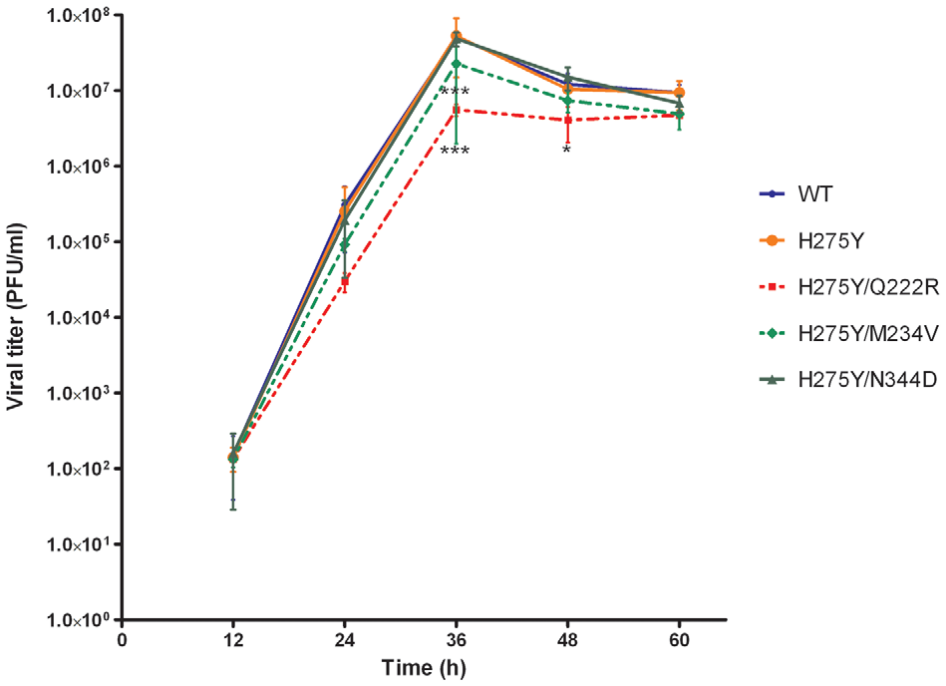
Replication kinetics of recombinant A/Brisbane/59/2007-like viruses *in vitro*. Confluent ST6Gal1-MDCK cells were infected with recombinant viruses at a multiplicity of infection (MOI) of 0.001 PFU/cell. Supernatants were harvested at 12 h, 24 h, 36 h, 48 h and 60 h post-infection and titrated by standard plaque assays. The mean values for three experiments with standard deviations are presented. *P<0.05 and ***P<0.001 for differences in viral titers when compared to the recombinant WT virus.

Viral fitness of recombinant A/Brisbane/59/2007-like viruses was assessed *in vitro* using ST6Gal1-MDCK cells. The double H275Y/Q222R mutant produced viral plaques with a significantly reduced area compared to the recombinant WT (0.13 mm^2^ vs 0.53 mm^2^, *P<0.001*) whereas the remaining recombinants generated plaques of comparable sizes (Table 1). Of note, the reduction in plaque size for the H275Y/Q222R mutant was also significant compared to that of the single H275Y mutant (*P<0.001*). In replication kinetics experiments, the peak viral titers for all recombinants were obtained at 36 h post-infection (PI) with viral titers ranging from 5.6×10^6^ PFU/ml (H275Y/Q222R) to 5.3×10^7^ PFU/ml (WT) (Fig. 3). The WT, the single (H275Y) and the double (H275Y/N344D) mutants had comparable viral titers at all time points. By contrast, and in accordance with plaque size data, the double H275Y/Q222R mutant was associated with a significant reduction in viral titers at 36 h (*P<0.001*) and 48 h (*P<0.05*) PI compared to the WT (Fig. 3). There was also a significant reduction in the viral titer obtained at 36 h PI for the double H275Y/M234V mutant compared to the WT (*P<0.001*). When compared to the single (H275Y) mutant, viral titers of the double H275Y/Q222R and H275Y/M234V mutants were significantly lower at 36 h (*P<0.001*).

**Figure 4:**
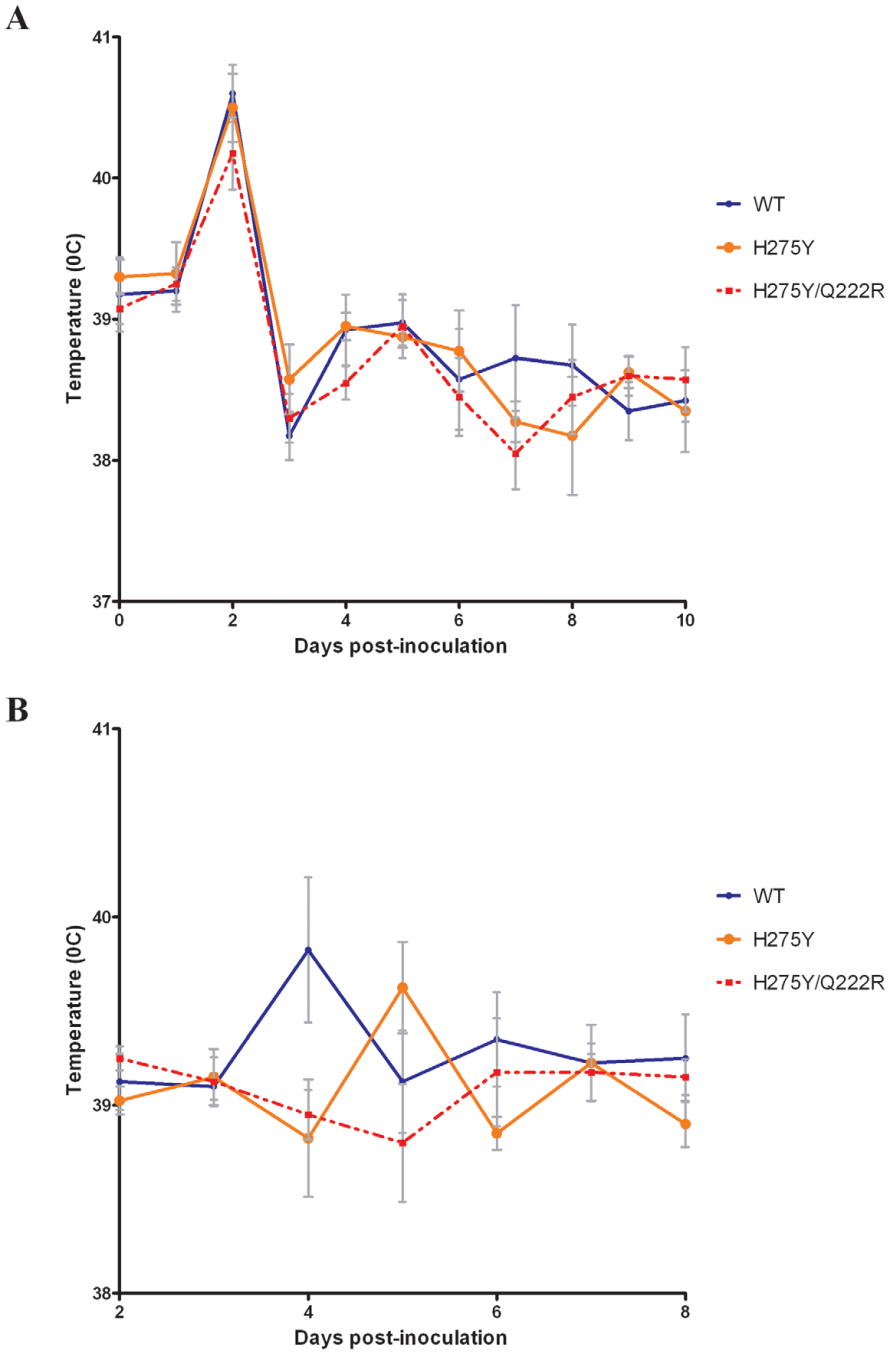
Body temperatures of infected and contact ferrets. Body temperatures were recorded by rectal thermometer during 10 days post-inoculation in groups of 4 index ferrets infected with 1.25×10^5^ PFU of recombinant A/Brisbane/59/2007-like wild-type (WT) virus as well as H275Y and H275Y/Q222R mutants (A) and in groups of 4 naïve ferrets that were placed in direct contact with index ferrets 24 h later (B).

**Figure 5:**
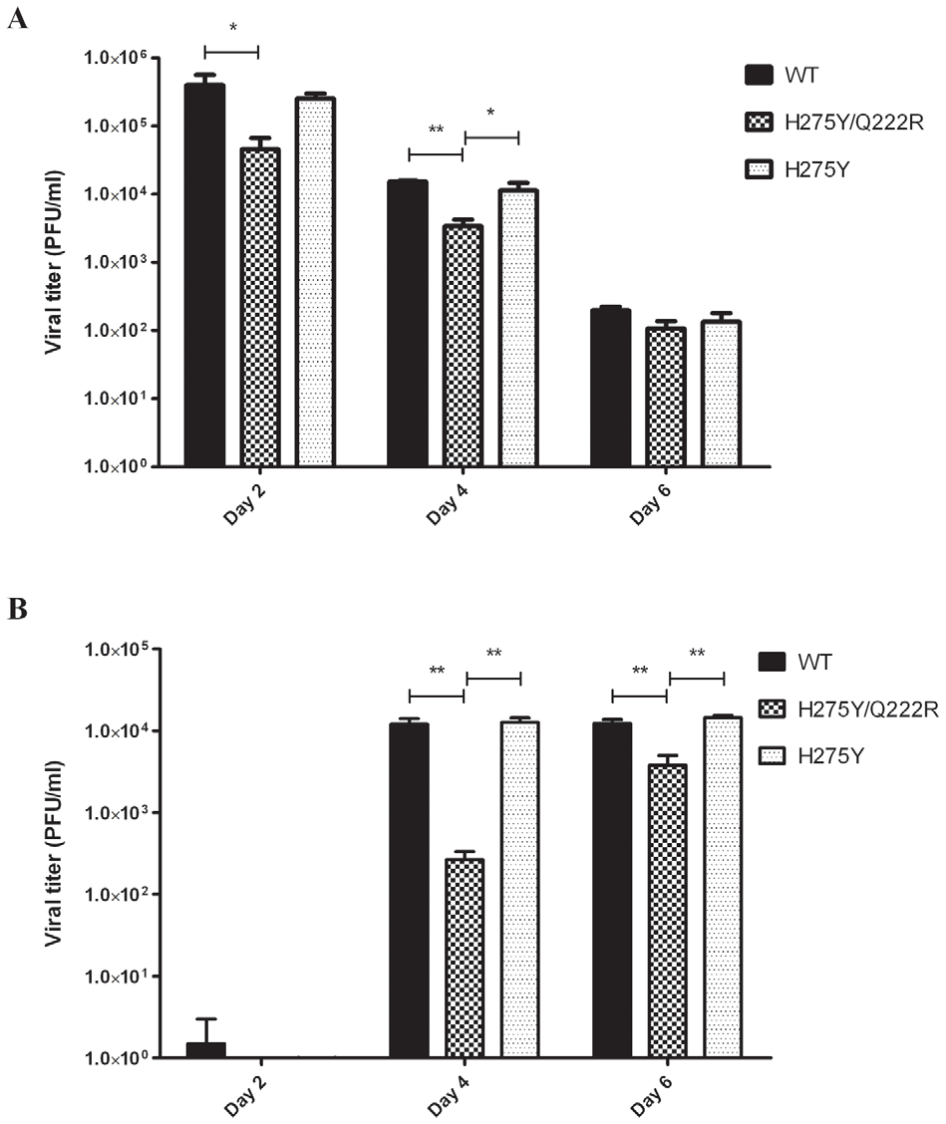
Mean viral titers in nasal wash samples of infected and contact ferrets. Mean viral titers ± standard deviations were determined in nasal washes by using standard plaque assays in groups of 4 index ferrets infected with 1.25×10^5^ PFU of recombinant A/Brisbane/59/2007-like wild-type (WT) virus as well as H275Y and H275Y/Q222R mutants (A) and in groups of 4 naïve ferrets that were placed in direct contact with index ferrets 24 h later (B).*P<0.05 and **P<0.01 for differences in viral titers when compared to the recombinant WT virus.

Intranasal inoculation of ferrets with the WT and two mutant (H275Y and H275Y/Q222R) Bris07 recombinant viruses resulted in a febrile response that peaked on day 2 PI (Fig. 4A). The area under the curve (AUC) of temperatures between days 0 and 6 PI was similar for the 3 groups of ferrets i.e. 6.81±1.19 for the WT virus, 5.99±1.9 for the H275Y/Q222R mutant and 7.26±0.55 for the H275Y mutant. There was no significant difference in body weight between the three groups of animals at any time points (data not shown). As shown in Fig. 5A, mean viral titers in nasal wash samples collected on day 2 PI from ferrets infected with the recombinant WT and the single H275Y mutant were comparable (4×10^5^±2.9×10^4^ PFU/ml for the WT and 2.6×10^5^±8.7×10^4^ PFU/ml for the H275Y mutant) whereas the H275Y/Q222R mutant had a reduced mean viral titer (4.6×10^4^±4.2×10^3^ PFU/ml; *P<0.05* vs WT). Similarly, mean viral titers in nasal wash samples of ferrets infected with the H275Y/Q222R were significantly lower than those of the H275Y mutant (*P<0.05)* and WT virus (*P<0.01)* on day 4 PI (3.4×10^3^±1.7×10^3^, 1.1×10^4^±6.7×10^3^ and 1.5×10^4^±9.6×10^2^ PFU/ml, respectively). On the other hand, the three recombinants were associated with comparable mean viral titers on day 6 PI (2×10^2^±4.6×10^1^ PFU/ml for the WT, 1.1×10^2^±5.8×10^1^ PFU/ml for the H275Y/Q222R and 1.3×10^2^±8.1×10PFU/ml for the H275Y).

All contact ferrets seroconverted for A/Brisbane/59/2007 when tested 14 days after contact, with geometrical mean hemagglutination inhibition (HAI) titers of 160±33, 145±119 and 95±55 for the WT, H275Y and H275Y/Q222R recombinant viruses, respectively. A febrile response could be observed on days 4 and 5 in the WT and the H275Y groups, respectively, but not in the H275Y/Q222R group (Fig. 4B). The AUC of temperatures between days 2 and 6 PI was similar between groups of ferrets infected with the recombinant WT (5.29±0.34) and its H275Y variant (4.54±0.19) whereas the AUC of the H275Y/Q222R group was significantly lower than that of the WT group (4.09±0.96; P<0.05). Viral titers in nasal wash samples collected on days 2, 4 and 6 PI are shown in Fig. 5B. Only the WT virus was detected on day 2 PI. Mean viral titers were comparable for the H275Y mutant and the WT virus on days 4 and 6 PI. In contrast, the H275Y/Q222R mutant was associated with significantly lower mean viral titers compared to WT on both day 4 (2.7×10^2^±1.2×10^2^ vs 1.2×10^4^±3.5×10^3^ PFU/ml, *P<0.01)* and day 6 PI (3.8×10^3^±2.1×10^3^ vs 1.2×10^4^±2.5×10^3^ PFU/ml, *P<0.01)*.

## Discussion

In this study, we used recombinant viruses derived from a clinical WT Bris07 strain to demonstrate using both *in vitro* and ferret experiments that the R222Q NA mutation was the main but possibly not the only permissive mutation that allowed the widespread dissemination of the oseltamivir-resistant H275Y mutant during the 2007–09 influenza seasons. Although such mutant seems to have disappeared since the emergence of the pandemic H1N1 virus in April 2009, understanding the mechanisms leading to the transmission of this unique virus is of great importance and could have an impact on the future use of NAIs.

The influenza NA protein plays a major role during the viral replication cycle. Its sialidase activity promotes virion release by removing sialic residues from viral glycoproteins and infected cells [14]. The NA enzyme also mediates virus penetration in the mucin layer of the respiratory tract, facilitating virus spread [15]. Importantly, the catalytic site of the NA enzyme has been shown to be conserved in all influenza A subtypes and influenza B viruses [3]. Therefore, the influenza NA protein has been considered as a suitable target for designing anti-influenza agents for both prophylactic and therapeutic purposes. Besides its functional role, the NA protein is a major structural surface glycoprotein that is exposed to the host immune pressure [14]. The NA gene, like the HA one, is therefore subject to more genetic variations than the rest of the influenza genome. Consequently, some amino acid (a.a.) changes, part of antigenic sites of the NA protein, may significantly contribute to the emergence of drifted variants, whereas certain substitutions located in or near the catalytic site may also affect the NA enzyme properties. For instance, Hensley and colleagues have recently identified NA mutations conferring resistance to zanamivir in variants of an influenza A/Puerto Rico/8/1934 H1N1 virus that was subjected to anti-HA monoclonal antibodies pressure [16].

In this study, we focused on a.a. changes that occurred in the NA protein during the evolution of recent seasonal influenza H1N1 viruses and that may have been involved in the development and dissemination of resistance to NAIs. These changes included the well-known framework H275Y mutation, responsible for the resistance phenotype to oseltamivir and peramivir, as well as other substitutions (V234M, R222Q and D344N) that may have contributed to the emergence and dissemination of resistance by acting as permissive/compensatory mutations.

Phylogenetic analyses previously demonstrated that the V234M mutation was already present in oseltamivir-susceptible A/Solomon Islands/3/2006 (SI06) viruses [13]. In another report, NA enzyme properties of SI06 viruses were found to be similar to those of older oseltamivir-susceptible strains such as A/New Caledonia/99/2001 in terms of relative NA activity (*Vmax*) and affinity (*Km*) [11]. By contrast, the appearance of the R222Q and D344N mutations in H1N1 viruses isolated after 2007 was associated with a significant increase in NA affinity (decreased *Km* values) in both 275H and 275Y strains [11]. In accordance with these observations, we demonstrated a sharp impact for the Q222R and N344D reversion mutations on *Km* values using our Bris07 recombinants (Table 1). Besides its effect on NA affinity, the Q222R reversion mutation was also associated with a significant decrease in relative NA activity (Table 1 and Fig. 1) and total NA activity that was expressed on the cell surface (Fig. 2), in line with previously-reported results in another viral background [13]. As a result, the H275Y/Q222R mutant virus was significantly compromised *in vitro* based on plaque size and replication kinetics patterns. Such decreased viral replication of the H275Y/Q222R mutant was also evident *in vivo*, resulting in lower viral titers in nasal wash samples and an absence of febrile response in contact ferrets. However, the H275Y/Q222R mutant was transmitted to all naïve ferrets by direct contact meaning that the combination of several permissive NA mutations and/or mutations elsewhere in the viral genome may be necessary to recapitulate the epidemiological observations showing increased transmission of the oseltamivir-resistant Bris07 virus. Also, it should be noted that naïve (non-immune) ferrets may not completely capture the fitness of Bris07 in humans with pre-existing immunity. Alternatively, the Q222R mutation could affect airborne transmission which has not been evaluated in our study. Of note, possibly due to the lower affinity of Q222R for MUNANA, less NAIs were required for competitive inhibition of the H275Y/Q222R mutant compared to the H275Y mutant. Residue 222 is located in the vicinity of the catalytic site of the N1 enzyme based on 3-D structure analysis [17]. Thus, substitution of a charged (R) by an uncharged (Q) a.a. at codon 222 may be the main change that dramatically altered the NA enzyme properties of recent seasonal H1N1 viruses. Of interest, only one NA substitution (R194G) was sufficient to restore the viral fitness of an influenza A/WSN/33 (H1N1) virus containing the compromising H275Y NA mutation [13].

In addition to the R222Q mutation, a permissive role was also suggested for V234M and D344N substitutions [11], [13]. Interestingly, in a recent report on the evolution of influenza NA genes, positive epistasis (i.e. combination of mutations that are substantially more beneficial than single mutations alone) was detected in pairs of codons within the NA gene of the N1 subtype including 275−222, 275−234, and 275−344 [18]. In our study, although the M234V and N344D reversions were associated with decreased relative NA activity and affinity, respectively (Table 1 and Fig. 1), none of these mutations significantly altered the viral fitness *in vitro*. Nevertheless, a possible synergy between these mutations and Q222R cannot be completely excluded.

Our study revealed that the H275Y NA mutation was not deleterious to fitness in the Bris07 genetic context in contrast to older H1N1 strains. However, this mutant did not have a replicative advantage compared to the WT as suggested by epidemiological studies. Indeed, the recombinant WT virus and its H275Y variant demonstrated similar replication kinetics during *in vitro* experiments. In addition, these recombinants had comparable infectivity and contact transmissibility in ferrets. Thus, the presence of the permissive mutations (R222Q, V234M and D344N) in the NA protein of our WT strain was apparently not sufficient to alter the viral fitness to the level that a compensatory change, such as the H275Y mutation, would be necessary. Therefore, we believe that changes in the NA gene alone may not provide a complete explanation for the emergence and spread of the oseltamivir-resistant H275Y Bris07 variant. Other changes in the genome might have been involved in this event. For instance, Yang and colleagues recently demonstrated that the dominant H275Y variant that emerged in Taiwan in 2007–2008 was a result of intra-subtypic reassortments between HA, NA, PB2 and PA genes from one clade (clade 2B) and the remaining 4 genes from another one (clade 1) [19]. Furthermore, the H275Y NA substitution and other changes in NA, HA, PB1 and PB2 proteins occurred in that background [19]. Thus, it would be also interesting to assess the effect of HA and particularly polymerase mutations that differed between WT and H275Y mutant clinical Bris07 isolates on replicative capacities and transmissibility.

Despite the fact that the secondary mutations described here were not investigated individually but in conjunction with H275Y, our study provides a comprehensive analysis of relevant permissive NA mutations in the contemporarily seasonal H1N1 background. This included *in vitro* characterization, assessment of viral fitness and contact transmission in ferrets as well as NA enzyme properties of recombinant mutants. In particular, our investigation clearly demonstrated the positive impact of one specific NA substitution (i.e. R222Q) in conjunction with the oseltamivir resistance H275Y mutation on enzymatic properties and viral fitness of the Bris07 H1N1 strain. Noteworthy, our results suggest that total NA activity was more likely predictive of *in vitro and in vivo* viral fitness than the enzyme affinity (Km) parameter. Whether the Q222R mutation is also deleterious in the absence of H275Y was not investigated here; however, in a previous work, influenza A/Paris/497/2007 (222Q/275H) and A/Solomon Islands/3/2006 (222R/275H) seasonal H1N1 isolates grew to comparable titers in *in vitro* kinetics experiments [11]. Although clinical 2009 pandemic H1N1 variants containing such permissive mutations have not been reported, a computational approach had recently led to the identification of R257K and T289M as potential secondary mutations in that context [20]. Thus, monitoring for resistance in influenza viruses should take into consideration not only NA resistance-mutations themselves but also permissive/secondary ones as the latter may significantly affect the clinical and epidemiological impacts of seasonal or pandemic influenza viruses.

## Materials and Methods

### Ethics Statement

All procedures were approved by the Institutional Animal Care Committee at Laval University according to the guidelines of the Canadian Council on Animal Care.

### Rescue of recombinant viruses

Reverse transcription-PCR using universal influenza primers [21] was used to amplify the eight genomic segments of an oseltamivir-susceptible A/Quebec/15230/08 (H1N1) isolate whose HA and NA genes shared respectively 99.53% and 99.71% nucleotide identity with those of the influenza A/Brisbane/59/2007 vaccine strain [10]. All segments were cloned into the pJET plasmid (Fermentas, Burlington, ON, Canada) and sequenced. Sequence analysis confirmed the presence of histidine (H), glutamine (Q), methionine (M) and asparagine (N) residues at residues 275, 222, 234 and 344 (N1 numbering), respectively, of the NA protein. The PB1, PB2 and PA segments were sub-cloned into pLLBG whereas the HA, NA, NP, M1/M2 and NS1/NS2 segments were sub-cloned into pLLBA bidirectional expression/translation vectors as described [22]. The pLLBA plasmid containing the NA gene was used for the introduction of the H275Y mutation using appropriate primers and the QuikChangeTM Site-Directed Mutagenesis kit (Stratagene, La Jolla, CA). The resulting pLLB-NA_275Y_ mutant plasmid was then used for reverting potential compensatory mutations (Q222R, M234V or N344D) as described above. All recombinant plasmids were sequenced to confirm the absence of undesired mutations. The eight bidirectional plasmids were cotransfected into 293T human embryonic kidney cells using the LipofectamineTM 2000 reagent (Invitrogen, Carlsbad, CA) as previously described [23]. Supernatants were collected 72 h post-transfection and used to inoculate ST6Gal1-MDCK cells kindly provided by Dr. Y. Kawaoka, University of Wisconsin, Madison, WI). The recombinant wild-type (WT) and H275Y, H275Y/Q222R, H275Y/M234V and H275Y/N344D mutant viruses were subsequently sequenced and titrated by standard plaque assays in ST6Gal1-MDCK cells.

### NA enzyme kinetics assays

A fluorometric based assay using MUNANA (Methylumbelliferyl-N-acetylneuraminic acid) (Sigma, St-Louis, MO) as substrate was performed to determine total NA enzymatic activity per infectious virus [24]. Briefly, recombinant viruses were standardized to an equivalent dose of 10^6^ plaque forming-units (PFU)/ml and incubated at 37°C in 50-µl reactions with different concentrations of MUNANA. The final concentration of the substrate ranged from 0 to 3000 µM. Fluorescence was monitored every 90 s for 53 min (35 measures). The Michaelis-Menten constant (*K_m_*) and the relative NA activity (*V_max_*) were calculated with the Prism software (GraphPad, version 5), by fitting the data to the Michaelis-Menten equation using nonlinear regression [25].

### Cell surface NA activity

Recombinant NA plasmids and pCAGGS-PA, -PB1, -PB2 and -NP plasmids were used to co-transfect 293T cells in order to express recombinant NA enzymes [26]. Twenty-four hours after transfection, the cells were briefly treated with trypsin-EDTA and neutralized by the addition of serum followed by centrifugation at 3000 RPM for 5 min. After washing twice with PBS, the cells were resuspended in a non-lysing buffer (15 mM MOPS, 145 mM sodium chloride, 2.7 mM potassium chloride and 4 mM calcium chloride, adjusted to pH 7.4) and used in an NA assay using the MUNANA substrate [13].

### NA inhibition assays

The drug resistance phenotype was determined by NA inhibition assays using the MUNANA substrate as previously described [26], with minor modifications. Briefly, recombinant viruses were standardized to a NA activity ten-fold higher than that of the background and then incubated with serial three-fold dilutions of the drugs (final concentrations ranging from 0 to 1800 nM), including oseltamivir carboxylate (Hoffmann-La Roche, Basel, Switzerland), zanamivir (GlaxoSmithKline, Stevenage, UK) and peramivir (BioCryst, Birmingham, AL). The 50% inhibitory concentration (IC_50_) was determined from the dose-response curve.

### *In vitro* replication kinetics experiments

Replicative capacities of the recombinant viruses were evaluated by infecting ST6Gal1-MDCK cells with a multiplicity of infection (MOI) of 0.001 plaque-forming units (PFUs)/cell. Supernatants were collected every 12 h until 60 h PI and titrated by plaque assays. The mean viral plaque area of recombinant viruses was determined from a minimum of 16 plaques obtained after 60 h of incubation under agarose overlay using the ImageJ software (version 1.41), developed by Wayne Rasband of the National Institutes of Health as previously described [25].

### Ferret studies

Groups of 4 seronegative (900–1500 g) male ferrets (Triple F Farms, Sayre, PA) were lightly anesthetised by isoflurane and received an intranasal instillation of 1.25×10^5^ PFUs of the recombinant Bris07-like WT, H275Y or H275Y/Q222R variants. Temperature of ferrets was measured by rectal thermometers every day until day 10 PI. Ferrets were weighed daily and nasal wash samples were collected from animals on days 2, 4 and 6 PI. Virus titers from nasal wash samples were determined by plaque assays using ST6Gal1-MDCK cells. Serum samples were collected from each ferret before intranasal infection and on day 14 PI to evaluate specific antibody levels against the seasonal Bris07 strain using standard HAI assays. To evaluate contact-transmissibility, inoculated-contact animal pairs were established by placing a naïve ferret into each cage 24 h after inoculation of the index ferret [27]. Contact animals were monitored for clinical signs and nasal wash and serum samples were collected as described above for determination of viral titers and serological status, respectively.

### Statistical analyses

NA kinetic parameters (*K_m_* and *V_max_* values), NAI IC_50_ values and viral titers *in vitro* and in nasal washes of ferrets were compared by one-way ANOVA analysis of variance, with the Tukey's multiple comparison post test. The amount of NA activity on the cell surface and plaque sizes of the recombinants were compared to those of the WT virus and/or the H275Y mutant by the use of unpaired two-tailed *t* tests.
